# *Cryptosporidium* spp., prevalence, molecular characterisation and socio-demographic risk factors among immigrants in Qatar

**DOI:** 10.1371/journal.pntd.0007750

**Published:** 2019-10-29

**Authors:** Sonia Boughattas, Jerzy M. Behnke, Duaa Al-Sadeq, Ahmed Ismail, Marawan Abu-Madi

**Affiliations:** 1 College of Health Sciences, Biomedical Research Center, Qatar University, Doha, Qatar; 2 School of Life Sciences, University of Nottingham, Nottingham, United Kingdom; 3 Medical Commission, Ministry of Public Health, Doha, Qatar; University of Iowa, UNITED STATES

## Abstract

**Background:**

The World Health Organization WHO has estimated that in developed countries, up to 30% of the population may suffer from foodborne diseases each year, and that in developing countries up to 2 million deaths per annum can be attributed to cryptosporidiosis. Reports have already emphasized the role of immigrants in outbreaks of parasitic diseases especially those working in food processing industries.

**Methodology/Principal findings:**

Herein we assessed *Cryptosporidium* spp. infections among immigrants in Qatar with a special focus on food handlers and housemaids. The overall prevalence of *Cryptosporidium* spp. by q-PCR among 839 asymptomatic subjects was 4.5%. Based on the Gp60 gene, the majority of isolates were identified as *C*. *parvum* subtype IIdA20G1b. The positive sample for *C*. *hominis* was subtyped as IeA12G3T3. Seven mixed infections were also identified (four *C*. *parvum* + *C*. *hominis*, and three *C*. *parvum* + *C*. *meleagridis*). The prevalence of *Cryptosporidium* spp. did not differ significantly between the sexes or age classes but varied significantly between subjects affiliated to different religions with the lowest prevalence among the Muslims. Multifactorial analysis retained also marked significance with education, income, and a house contents index.

**Conclusions/Significance:**

Our results contribute to a better understanding of the epidemiology of cryptosporidiosis and the risk factors associated with the likelihood of carrying this infection among immigrant workers from developing countries.

## Author summary

Cryptosporidiosis is a diarrheal disease caused by the parasite *Cryptosporidium* with the potential of morbidity and mortality among fragile subjects. Although the frequency of this parasite is higher in under-developed countries, it can be a frequent source of diseases, in developed areas. This may be due, among other factors, to the immigration flows from poor endemic places to industrialized countries contributing thus to the parasite spread. Human illness was formerly thought to be caused by a single *Cryptosporidium* species, but advanced studies have demonstrated that it is caused by more than 20 different species. We examined characteristics and risk factors associated with *Cryptosporidium* among new immigrants in Qatar. We predominantly identified *Cryptosporidium parvum* as infecting species. We found that factors like religion, education, monthly income and the house contents play a major role in the parasite occurrence. Therefore, there is a need for better awareness about the parasite and about strategies for its eradication among the new immigrants in Qatar.

## Introduction

Cryptosporidiosis is recognized as one of the leading causes of diarrhoea with dramatic adverse effects resulting in mortality especially among children [1, 2]. Although the prevalence of this protozoan is higher in under-developed countries, cryptosporidiosis is also a frequent source of diseases, in developed/ industrialized parts of the world. The Center for Diseases Control and Prevention (CDC) reported recently that the number of *Cryptosporidium* cases in the United States of America appears to be on the rise; there were 32 outbreaks of *Cryptosporidium* reported in 2016 compared to 16 in 2014 [3]. Intestinal protozoan infections in developed countries are in continuous rise due to globalization of the food supply and immigration from poor endemic regions to more affluent parts of the world [4]. Growth in the economies of developed and developing countries, with rapidly expanding industrialization, has resulted in a demand for a bigger labour force. Hence foreign workers from low-wage countries have been encouraged to migrate to fill the gaps, motivated by an understandable drive to improve their own and their families’ standards of living. Such economic migration affects public health policies [5].

Because of the ubiquitous presence of *Cryptosporidium*’s oocysts in the environment, humans can acquire *Cryptosporidium* infections through several transmission routes, including direct contact with infected persons (person-to-person transmission) or animals (zoonotic transmission); and ingestion of contaminated food (foodborne transmission) or water (waterborne transmission) [6]. However, in Qatar, little contact with farm animals is observed and the country relies on desalinated sea water that is piped to houses and used for drinking; thus these two potential routes for transmission are unlikely within the country. The foodborne route is more likely to play a role in transmission because of importing fresh vegetables from other countries. This globalization of the food supply has indeed increased the chances of parasite transmission from food producing countries [7]. Person to person transmission is also a possible route for transmission in Qatar [8]. However, immigrants to Qatar originate from various parts of the world where all four transmission routes may be relevant, and despite the introduction of the pre-employment certificates based on a medical examination in their country of origin, enteric helminth and protozoan infections are still identified regularly in newly arrived immigrant workers examined at the Medical Commission to which they have to report soon after arrival to obtain work permits [9].

Immigrants engaged in some jobs may be particularly at risk of infection and thus a source of infection for others in the community. Food handlers and housemaids are good examples because of their close contact with food that may be contaminated or that they may contaminate, if already infected themselves and unaware of the routes and risks of transmission, or simply careless in their hygiene. A survey in Oman among mostly Indian food handlers revealed that 12.7% were infected with intestinal parasites [10]. With a lack of treatment and/or poor personal hygiene habits including failure to wash hands, food handlers can cause faecal contamination of foods during preparation leading to immense consequences for consumers [11]. The CDC has emphasized that poor personal hygiene is the third most commonly reported food preparation practice contributing to foodborne disease [12]. A survey conducted by the Food and Agriculture Organization in 2001 recorded a higher incidence of foodborne illnesses in areas of increased food vendors activity [13].

Recent advances in knowledge of the genetic structure of *Cryptosporidium* spp. had a marked impact on our understanding of the epidemiology of this parasite and the public health significance of the different species and their genotypes [14]. The aim of this study was first to identify the species of *Cryptosporidium* and their genotypes among immigrant workers to Qatar, focusing to some extent on food handlers and housemaids because of their potential to pass infections to consumers and households in which they work, respectively. We assessed the prevalence of *Cryptosporidium* infections in this subset of the population, among those in different job families; and related prevalence of *Cryptosporidium* to a range of intrinsic and extrinsic factors that were quantified in our effort to understand the epidemiology of this parasite among recently arrived immigrant workers.

## Methods

### Study population

The study population has already been fully described elsewhere [9] and we followed an identical procedure here. The subjects, who came from 16 countries, were allocated to four age classes, and to four regions of origin: eastern Asia (*n* = 175), western Asia (*n* = 612), northern and saharan Africa (*n* = 18) and sub-saharan Africa (*n* = 34). Samples were processed as described in details previously [9] and subjected to DNA extraction using the QIAamp DNA stool minikit (Qiagen, Hilden, Germany), according to the manufacturer’s instructions.

### Ethics statement

All the adult subjects provided written informed consent. The Ethical approval was obtained from the Medical Research Centre and Research Committee at Hamad Medical Corporation, Qatar (Research protocol # 16367/16 (NPRP8–1556–3–313)).

### Molecular analysis

*Cryptosporidium* diagnosis was achieved by quantitative PCR (qPCR) (Applied Biosystems Cycler 7500) using Hot Start Taq *Plus* master mix kit (Qiagen, Hilden, Germany) in a total reactional volume of 20 including 3μl of DNA sample with 0.3μM SCL2 (CAGTTATAGTTTACTTGATAATC) as forward primer, 0.3μM SCR2 (CAATACCCTACCGTCTAAAG) as reverse primer and 0.03μM CrySB (FAM/CCGTGGTAATTCTAGAGCTA/BHQ) as a probe targeting a 214bp fragment of 18S rRNA gene. The qPCR cycling was initiated by 95°C for 10min activation step, followed by 50 cycles each cycle comprising 95°C for 15 s, 50°C for 30 s, and 72°C for 45 s each, and a final extension at 72°C for 2 min [15].

The qPCR positive products for *Cryptosporidium* spp. were subjected to restriction fragment length polymorphism (RFLP) analysis to identify the *Cryptosporidium* species with the endonucleases AseI, Taq1 and MseI. The enzymatic digestion products were run on agarose electrophoresis and species assignment was carried out by comparing RFLP profiles to those reported previously [16].

Gp60 gene fragment was amplified by nested PCR (nPCR) to subtype *Cryptosporidium parvum* and/or *Cryptosporidium hominis* isolates [17]. The nPCR products were purified and subjected to sequencing at MCLAB facilities (Molecular Cloning Laboratories- San Francisco, USA). Sequences were aligned and edited using BioEdit Software and consensus sequences were then scanned against the GenBank database using BLAST to determine the similarity of the isolates to already published sequences [18]. Gp60 subtypes of *Cryptosporidium* are named according to the repeats number of ‘‘A” (TCA), ‘‘G” (TCG), ‘‘T” (TCT): The difference between the repeats number will differentiate the multiple subtypes of each species. The Gp60 nucleotide sequences of the different subtypes were deposited in the GenBank database and the accession numbers are provided in the results section.

### Data sources

Information obtained at interviews, when collecting the samples, was first recorded on hard copies of printed pro-formas of a questionnaire. Subsequently, this was entered into an Excel workbook and quality controlled for accuracy and any missing values. Factors recorded on personal and familial relationships comprised; age, sex, religion, region of origin, immigration status, education, job/profession, and monthly income (Table 1).

**Table 1:** Prevalence (%) of *Cryptosporidum* spp. among immigrant workers to Qatar, according to the subjects’ personal characteristics, migration history, education, and job family. Statistical analysis was based on single factor models. For multifactorial analysis see text.

	*n*	No. infected Prevalence (%)	CL_95_	Odds ratio^β^	*X*^*2*^	*P*
**PERSONAL CHARACTERISTICS** **Age**						
**18–22 years**	110	2 (1.8)	0.57–5.07	0.019		
**23–29 years**	351	19 (5.4)	3.34–8.61	0.057		
**30–37 years**	229	11 (4.8)	3.15–7.19	0.050		
**38–56 years**	149	6 (4.0)	1.65–8.78	0.042	3.13	0.37
**Sex**						
**Male**	522	23 (4.4)	3.27–5.91	0.046		
**Female**	317	15 (4.7)	2.89–7.59	0.050	0.048	0.83
**Religion**						
**Buddhist**	15	1 (6.7)	0.35–30.20	0.071		
**Christian**	217	11 (5.1)	3.39–7.45	0.053		
**Hindu**	225	19 (8.4)	6.19–11.42	0.092		
**Muslim**	382	7 (1.8)	0.74–4.32	0.019	14.92	**0.002**
**Region of origin**						
**west Asia**	612	27 (4.4)	3.20–6.04	0.046		
**eastern Asia**	175	10 (5.7)	2.65–11.52	0.061		
**northern & saharan Africa**	18	0 (0)	0.00–18.52	0.000		
**sub saharan Africa**	34	1 (2.9)	0.35–13.05	0.030	2.44	0.49
**Education**						
**None**	194	2 (1.0)	0.08–5.29	0.010		
**Elementary school only**	466	33 (7.1)	4.31–11.32	0.076		
**Up to intermediate school**	33	0 (0)	0.00–8.04	0.000		
**Up to high school**	126	3 (2.4)	0.83–6.16	0.024		
**Graduate/postgraduate**	20	0 (0)	0.00–16.68	0.000	20.46	**<0.001**
Occupation/Profession*						
**Blue collar**	419	21 (5.0)	2.88–8.50	0.053		
**Pink collar**	42	0 (0)	0.00–9.69	0.000		
**White collar**	15	0 (0)	0.00–22.22	0.000		
**Housemaids**	308	15 (4.9)	3.02–7.71	0.051		
**Food handlers**	55	2 (3.6)	1.12–9.69	0.038	5.69	0.22
**Monthly income (QR)**						
**600–999**	209	3 (1.4)	0.68–2.96	0.015		
**1000–1499**	383	22 (5.7)	3.51–9.20	0.061		
**1500–2999**	218	13 (6.0)	4.10–8.53	0.063		
**>2999**	29	0 (0)	0.00–11.53	0.000	11.09	**0.011**

^**β.**^ The values of the Odds Ratios provided reflect the likelihood of being infected compared to being without infection at each level within the factors listed. A value of 1 would reflect equal likelihood of either being infected or without infection, i.e. a prevalence of 50%.

*Occupation/Profession:

Blue collar: mechanics, masons, builders, car wash attendants, carpenters, cleaners, crane operators, drivers, electricians, fire fighters, fitters, gardeners, labourers, painters, plumbers, steel fixers and welders.

Pink collar: barbers, beauticians, butlers, grocers, hairdressers, lifeguards’ merchandisers, nurses, safety officers/guards, salespersons, saloon workers, security guards and tailors.

White collar: accountants, cashiers, civil engineers, clerks, IT experts, office boys, receptionists, and secretaries.

Food handlers: bakers, butchers, chefs, cooks, kitchen assistants, waiters/waitresses.

The household contents index was based on 1 point for each of the following: gas or electricity cooker, microwave oven, fridge, television, radio, computer, internet access, shower, bath, and car. We asked also about the provision and treatment of household water and toilet facilities and, whether the subject was a farmer. The number of animal species was based on a choice from dog, goat, cow, cat, chicken and other, and 1 point was given for each species (Table 2).

**Table 2:** Prevalence (%) of *Cryptosporiduim* infection based on factors in the country of origin.

	*n*	No. infected Prevalence (%)	CL_95_	Odds ratio	*X*^*2*^	*P*
**House contents Index**						
**0**	209	6 (2.9)	1.67–4.76	0.030		
**1**	215	22 (10.2)	7.72–13.33	0.114		
**2**	325	5 (1.5)	0.63–3.64	0.016		
**3**	55	4 (7.3)	3.35–14.64	0.078		
**4**	21	1 (4.8)	0.25–23.26	0.050		
**5–10**	14	0 (0)	0.00–23.81	0.000	24.66	**<0.001**
**Toilet**						
**Flushing**	99	1 (1.0)	0.06–9.01	0.010		
**Pit latrine**	732	37 (5.1)	3.64–6.94	0.053		
**None**	8	0 (0)	0.00–36.46	0.000	4.54	0.103
**Provision of household water**						
**None**	7	1 (14.3)	0.74–55.42	0.167		
**Inside tap**	593	31 (5.2)	3.90–6.92	0.055		
**Outside tap**	18	1 (5.6)	0.29–27.13	0.059		
**Shared tap**	19	1 (5.3)	0.27–25.70	0.056		
**Covered well**	31	0 (0)	0.00–7.67	0.000		
**Uncovered well**	126	2 (1.6)	0.42–5.03	0.016		
**Borehole**	3	0 (0)	0.00–63.15	0.000		
**River**	29	2 (6.9)	1.24–22.07	0.074		
**Bottled water**	13	0 (0)	0.00–22.51	0.000	9.72	0.285
**Treatment of drinking water**						
**None**	816	38 (4.7)	3.25–6.60	0.049		
**Boiling**	7	0 (0)	0.00–37.71	0.000		
**Filtration**	16	0 (0)	0.00–20.83	0.000	2.16	0.339
**Farmer cultivates food**						
**No**	722	34 (4.7)	3.37–6.53	0.049		
**Yes**	116	4 (3.4)	1.49–7.31	0.036	0.39	0.530
**Domestic animals**						
**Kept**	639	33 (5.2)	3.81–6.92	0.054		
**None kept**	200	5 (2.5)	1.48–6.73	0.026	2.83	0.092

### Statistical analysis

Prevalence values (percentage of subjects infected, based on presence/absence of *Cryptosporidium* spp. and hence binomially distributed data) are given with 95% confidence limits [CL_95_, in square brackets in the text], calculated by bespoke software [19]. Analysis of data was undertaken in two phases because of the number of potential explanatory factors recorded in the questionnaire. First, we fitted individual log-linear models (IBM Corp. Released 2011. IBM SPSS Statistics for Windows, Version 20.0. Armonk, NY: IBM Corp.) for each of the personal characteristics and then for each factor in the country of origin, and infection (presence/absence of *Cryptosporidium* spp.), as described fully earlier [9]. Then selecting only the factors that had been identified as significant in this initial phase, we repeated the procedure, this time fitting all significant factors (personal and from the country of origin) in a single multifactorial model. Model simplification was by backward selection, until only significant effects remained.

We also used the Chi Squared test to assess how infected and non-infected subjects were distributed among the various levels within each factor, and whether the two categories differed in this respect. The non-parametric Spearman’s test was used to assess correlations between continuous scaled variables. We tested co-occurrence of species by the null model of Janovy *et al* [20] based on parasite species density distributions in an assemblage.

## Results

### Genotype and subtype analysis of *Cryptosporidium*

The overall prevalence of *Cryptosporidium* spp. among the 839 subjects was 4.5% [CL_95_ = 3.12–6.48]. All of the 38 samples that were positive by qPCR, were successfully genotyped to determine the *Cryptosporidium* species. The PCR-RFLP analysis of 18S rRNA revealed distinctive banding patterns. Three species were identified: *C*. *parvum*, *C*. *hominis* and *C*. *meleagridis*. Thirty-seven subjects were infected with *C*. *parvum* among which seven had concurrent infections: four were mixed *C*. *parvum* + *C*. *hominis*, and three mixed *C*. *parvum* + *C*. *meleagridis*. There was also one case of a solo infection with *C*. *hominis*. Based on the observed prevalence of each species [20] (null model of co-occurrence of species), there were more concurrent infections than expected based the individual prevalence of each species in the study population (χ^2^ = 108.7, *P*<0.001).

**Fig 1:**
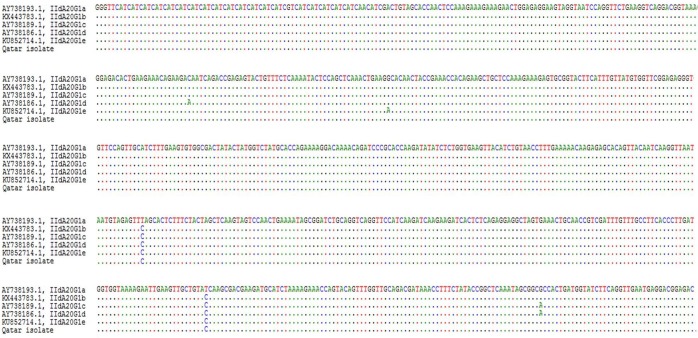
Multiple alignment analysis of Gp60 nPCR product sequences. Sequences are from the 5 IIdA20G1 subtypes a, b, c, d and e, and one Qatari isolate. Periods (.) indicates identical nucleotides related to the sequence of IIdA20G1a (first row).

The Gp60 gene was successfully sequenced in all the thirty-one single *C*. *parvum* and *C*. *hominis* isolates. Using the nomenclature system, all the thirty single *C*. *parvum* isolates were classified among IId family and subtyped as IIdA20G1 based on the number of trinucleotide repeats [17]. Five SNPs are described within the IIdA20G1 subtype subdividing it to: IIdA20G1a, IIdA20G1b, IIdA20G1c, IIdA20G1d, IIdA20G1e subtypes. The Multiple Alignment analysis showed that our *C*. *parvum* subtype (Accession number: MH114009) clustered with the subtype identified as IIdA20G1b (Genbank KX443783.1) sharing the two “T” to “C” substitutions but clearly separated from IId A20G1a, c, d, e subtypes (Fig 1).

Regarding the *C*. *hominis* isolate, and following the same nomenclatures, we identified twelve repeats of (TCA), three repeats of (TCG) and three repeats of (TCT) classifying the subtype, isolated from a 40 years old Filipino man, as IeA12G3T3 (Accession number: MH114010).

### Prevalence of *Cryptosporidium* among different subsets of the study group

#### Personal

The prevalence of *Cryptosporidium* spp. (combined) did not differ significantly between the sexes or age classes (Table 1).

Prevalence did vary significantly between subjects affiliated to different religions (lowest among the Muslims and highest among the Hindus; Table 1). Comparing infected and non-infected subjects, there was little difference for Christians (28.9% and 25.7%) and Buddhists (2.6% and 1.7%, respectively). These differences in the distribution of people from different denominations among infected and no-infected groups were significant (*χ*^2^_3_ = 14.5, *P* = 0.0023).

#### Region of origin

There was no significant difference in prevalence between subjects from the four geographical regions (Table 1), but interestingly only one case came from an African immigrant (male Kenyan), and the remaining 37 were all from Asian subjects. Most cases were among subjects from Indian (*n* = 14) and the Philippines (*n* = 9).

There was a significant difference in prevalence between those who had arrived in Qatar for the first time, and were applying for their first residency and work permits, and those that had stayed previously and was applying for renewal of their permit. All 38 cases of *Cryptosporidium* spp. infection were among the first-time arrivals (χ^2^ = 4.4, *P* = 0.036).

#### Education, occupation/profession and income

Although no infections were detected among pink- and white-collar workers, the sample sizes for these job families were relatively small. There was no significant difference in prevalence between subjects from the five job families (Table 1).

Most of the infections were detected in subjects who had attended elementary schools and had not experienced any further education (33/38) and this difference in prevalence between the five levels of education was significant (Table 1). There was also a highly significant difference in the distribution of infected and non-infected persons among the five educational levels (*χ*^2^_4_ = 14.3, *P* = 0.0064; e.g. 86.8% of infected people only went to elementary school, while for non-infected subjects this value was 54.1%).

There was also a significant difference in the prevalence of *Cryptosporidium* spp. between subjects with different monthly incomes. Prevalence was low among those in the top earning category (Table 1). We tested additionally for a relationship between monthly earnings of subjects and the household contents index (see below) in their country of origin, and found that these were significantly correlated (*r*_*s*_ = 0.298, *n* = 839, *P*<0.001).

#### Factors in country of origin

Table 2 shows the analysis of factors that were considered for the residence of each subject in their country of origin. Only the house contents index proved to have a significant effect on prevalence, and as can be seen the highest prevalence was in households with one of the lowest values for the index, i.e. for those with just one of the ten items that were listed. Among non–infected subjects 49% were in categories 0 and 1 combined, whereas for infected subjects the figure was 74%. Overall there was also a highly significant difference in the distribution of infected persons among the six levels for the house contents index (categories 5–10 were combined because there were so few subjects) compared with non-infected people (*χ*^2^_5_ = 27.0, *P*<0.0001).

There was no significant difference in the prevalence of *Cryptosporidium* spp. between animal owners and those who did not keep animals, nor in the distribution of infected and non-infected subjects among those who did and did not keep domestic animals (*χ*^2^_1_ = 2.58, *P* = 0.11).

The vast majority (86.8%) of the people in this study lived in houses in their countries of origin with pit latrines, rather than flushing toilets or depending on the bush. Prevalence of *Cryptosporidium* spp. did not differ between the three toilet types (Table 2), but the percentage of infected subjects who depended on pit latrines was higher with 97.4% compared to 86.8% among the non-infected.

### Combined analysis of individual characteristics of subjects

Multifactorial analysis of the five significant individual factors above revealed that four retained marked significance (Education, *χ*^2^_4_ = 19.4, *P* = 0.001; Income, *χ*^2^_3_ = 8.67, *P* = 0.034; Religion, *χ*^2^_3_ = 13.74, *P* = 0.003; House contents Index, *χ*^2^_3_ = 19.57, *P* = 0.002), whereas immigration turned out just the wrong side of the cut-off for significance (*χ*^2^_1_ = 3.41, *P* = 0.065). There were no significant interactions between any of these factors.

## Discussion

In this study, we assessed *Cryptosporidium* infections among immigrant workers, registering for work permits in Qatar, with a particular focus on food handlers and housemaids because infected individuals in these occupations pose a particularly high risk of transmission to other members of the community [21]. We also included those registering for other job families in our survey.

Molecular epidemiological studies of cryptosporidiosis have helped us to gain a better understanding of the transmission of *Cryptosporidium* spp. and its public health significance in human, animals and the environment [22]. The discovery in recent years that not only are there different species of *Cryptosporidium* but also that each can exist in several distinct genotypes, and that there are important differences in their host specificities, potential for zoonotic transmission and pathogenicity [6], raises the first question as to which species/genotypes were present in our sampled population. Hence, knowledge of the *Cryptosporidium* species/subtypes infecting our immigrant workers is perhaps the most important initial step in risk management of these parasites and the control of diseases that may potentially spread in the country.

Our molecular analysis has revealed that the vast majority of the isolates (78.9%) were *C*. *parvum* and all of these conformed to the genetic signature of the IId subtype family. This is in concordance with our previous observations within a pediatric population with predominance of *C*. *parvum* IId subtype among hospitalized children [15]. The IId subtypes of *C*. *parvum* have never been found in humans nor calves from the United States of America and Canada [6]. This subtype family is known to preferentially infect sheep and goats rather than cattle [23, 24]. It has been frequently reported from humans in the Middle East [25] and Iran [14] with sporadic reports in Portugal, Ireland, the Netherlands and Australia [26]. The predominance of the IId subtype family of *C*. *parvum* in the Middle East suggests that animal-to human transmission may be a common transmission route of *Cryptosporidium* in this geographic area. However, according to previous studies investigating diarrheic children living in Qatar [15] and Kuwait [27], very limited, if indeed any, contact with farm animals has been recorded and since clean desalinated seawater is the major source of drinking water, waterborne transmission is also unlikely to be a major factor. Based on these studies, it seems that the source of *C*. *parvum* infection among the settled populations in Kuwait and Qatar is much more likely to be foodborne or through person to person contact [28, 29].

The infected subjects in the current survey were healthy individuals without any symptoms of enteric infection. They originated from Bangladesh, India, Indonesia, Kenya, Nepal, the Philippines, and Sri-Lanka. Yet very limited molecular heterogeneity among the identified *Cryptoporidium* subtypes was observed which at first sight may suggest a common origin of the infections taking place in Qatar. This hypothesis may be supported by the fact that subtype IIdA20G1 has been previously identified in other subsets of the population of Qatar [15]. All first-time arrivals in Qatar have to report to the Medical Commission within 3 months, and since the samples in the current study were obtained during their medical checkup, there was a window of opportunity for the subjects to acquire the infection after their arrival in the country. Currently, there are no available data on the molecular signature of *Cryptosporidium* subtypes in the majority of the countries from which the infected individuals originated. For example, reports from the Philippines investigating different environmental sources have not specified the involved subtype. However, with the recent identification of the subtype IIdA20G1 in Italy [18], Turkey [30], China [31] and elsewhere in Spain [KJ756204] and among travelers in Sweden [JQ028866], it would appear that this subtype may be more widely distributed than previously thought, so the potential of initial contamination from outside Qatar cannot be totally excluded.

Two other species were identified in our study, *C*. *hominis* and *C*. *meleagridis*. *C*. *hominis* is considered to be mainly a human-infecting species [6], and as with other species it comprises a number of subtypes. In our study only 5 of the infected subjects harboured this species and four of them were concomitantly infected with *C*. *parvum*. The single infected *C*. *hominis* isolate was subtyped as the IeA12G3T3 subtype, which is the most rarely identified subtype of this species. So far, it has been recorded only in China from HIV-positive patients [23] and raw wastewater [32]; in Jamaica from HIV-infected persons [33]; in Australia within a waterborne cryptosporidiosis outbreak [34]; in Tasmania among clinical cases of diarrhea [35], and in Slovakia from immunocompetent individual [36].

*Cryptosporidium meleagridis* is the third most common species involved in human cryptosporidiosis [37] and is the only *Cryptosporidium* species that can infect both birds and mammals. This species has been recorded in both immunocompromised and immunocompetent humans in industrialized and developing countries and was responsible for an outbreak of gastroenteritis in Japan [38]. This pathogen has been commonly reported within mixed infection [26] but its transmission is not yet fully understood.

The overall prevalence of *Cryptosporidum* in our study population was 4.5%, which at first sight does not appear to be an excessively high figure, and is similar to what reported elsewhere among foreign workers in Taiwan [39], Malaysia [40] and some neighbouring countries [41]. Nevertheless, given the potential of this parasite to cause life threatening diarrhoea in some patients [42], this value is of some concern for public health in Qatar. In order to gain some insight into the likely transmission routes, we also asked each subject to volunteer relevant information about themselves and their living conditions in their country of origin.

Socio-demographic and cultural factors are important in the introduction and spread of protozoa in communities where sanitary conditions and infrastructures are inadequate [43]. Analysis of the information supplied for our survey revealed that the prevalence of *Cryptosporidium* spp. did not differ significantly between the sexes, as reported for example from Eastern Cape Province of South Africa [44] or between age classes, as observed in Cambodia [45]. There was no difference in prevalence between immigrants from different regions of the world, although some regions were under-represented in our study but in general prevalence values appeared to be higher among immigrants from Asian countries.

However, prevalence differed significantly between subjects affiliated to the four religious’ groups. The lowest prevalence was recorded among the Muslims and this could be attributable to the routine washing after use of toilet facilities that is practiced by Muslims through their religious code of behaviour. Similar observations have been reported for hookworm infections in India [46]; whereas non-significant correlations were observed for protozoan infections in Thailand and Ethiopia [5, 47].

Perhaps the most important route of transmission of *Cryptosporidium* elsewhere is waterborne, via sources that have become contaminated with excrement containing oocysts from humans and animals. Waterborne outbreaks of cryptosporidiosis have been extensively reported and investigated [5, 45, 48, 49]. For this reason, we asked our respondents about the water supply to their house in their country of origin and about any treatment of water before consumption. While prevalence of *Cryptosporidium* was higher among those with no access to household water or those dependent on river water, and zero among those utilizing only bottled water, dependent on borehole water or a covered well; there were very few cases in all these categories and overall we found no significant difference in prevalence among the 9 categories that we included in the analysis. Most subjects did not treat water in any way, but among the few that boiled water or used filtered water, there were no cases of *Cryptosporidium*, but again there were insufficient cases in these categories to enable a robust analysis.

Our multifactorial model, which took into account all the significant factors from the first round of analysis, identified three as significant (education, income, and house contents index). Two of these factors were indicative of *Cryptosporidium* infections being more frequent among the poor sectors of the immigrant community: those who were poorly educated and living in housing with few possessions that reflect a degree of affluence.

The conditions in which people live and their standards of living are likely to have a major influence on the infections to which they are exposed, particularly in relation to inadequate infrastructure of housing. However, while in our study prevalence was higher among those living in households with pit latrines, this did not differ significantly with households equipped with flushing toilets or those without any toilet facilities. Other studies have indicated high infection rates associated with the use of pit latrine toilets in Venezuela [50]. Elsewhere a study examining latrines as a source and sink of environmental pollution reported strong evidence of contamination with protozoan cysts/oocysts [51].

The monthly income of a family obviously determines in a great measure their economic status and standard of living, as reflected for example in their living accommodation and house contents. A relatively high income should be associated therefore with a higher house content index. There were no cases of *Cryptosporidium* infection among the very top earners, even if they were underrepresented in our survey (only 29 individuals). This conclusion is consistent with some other studies as for example in the USA where the lowest prevalence of *Cryptosporidium* was seen among subjects with the highest monthly income [49]. Other studies, as for example in Iran and Ethiopia, have found also that subjects with lower earnings were more at risk of infection [47].

In this study, a significant association was found between *Cryptosporidium* prevalence and the educational status of the subjects. Prevalence of *Cryptosporidium* was associated with workers whose schooling was limited to the elementary school education level. Reduced prevalence was observed among subjects with higher educational levels. This result highlights the potential links between social marginalization and *Cryptosporidium* positivity as such groups may lack the knowledge necessary to avoid parasite exposure [52]. Educated people are more aware of the protozoan transmission and they may apply the necessary measurements to avert the infection [53]. These results reflect the complexity of the relationships between people’s living standards, education and risk of infection [49, 54]. Accordingly, increased and better health awareness among immigrants is crucial and an important requirement for the prevention and control of intestinal parasitic diseases in Qatar [8].

There are a number of limitations in our study. We focused in the current work on asymptomatic subjects so these may not fully represent the disease burden (which could have been even higher had symptomatic subjects been included) and risk factors for *Cryptosporidium*. Despite the substantial initial sample size, only 38 samples were found positive for *Cryptosporidium* and this might have limited the robustness of the statistical analysis, with some under-representation in subsets of the study group. Mixed infections were observed among 18% of the isolates which had limited the genetic diversity analysis especially for the unique isolate of *C*. *meleagridids*.

### Conclusion

This study is the first to describe the homogenous nature of *Cryptosporidium* species among asymptomatic immigrants in Qatar. *Cryptosporidium parvum* was the most common species with the subtype IIdA20G1. Multifactorial analysis revealed that only religion, education, income, and a house contents index were significantly related to prevalence. Knowledge of the *Cryptosporidium* species and an understanding of the risk factors is essential for cryptosporidiosis management, since only with an understanding of all the links in the chain, the transmission can be broken and thereby terminated. Education and awareness program should be implemented targeting immigrants about these infections and their modes of transmission.

## References

[1] Aldeyarbi HM, Abu El-Ezz NM, Karanis P. Cryptosporidium and cryptosporidiosis: the African perspective. Environ Sci Pollut Res Int. 2016; 23: 13811–13821. 10.1007/s11356-016-6746-6 27126869

[2] Arndt MB, Walson JL. Enteric infection and dysfunction—A new target for PLOS Neglected Tropical Diseases. PLoS Negl Trop Dis. 2018; 12: e0006906 10.1371/journal.pntd.0006906 30592716 PMC6310236

[3] CDC, 2017: https://www.cdc.gov/media/releases/2017/p0518-cryptosporidium-outbreaks.html

[4] Calderaro A, Montecchini S, Rossi S, Gorrini C, De Conto F, Medici MC, et al Intestinal parasitoses in a tertiary-care hospital located in a non-endemic setting during 2006–2010. BMC Infect Dis. 2014; 14: 264 10.1186/1471-2334-14-264 24886502 PMC4029911

[5] Sagnuankiat S, Wanichsuwan M, Bhunnachet E, Jungarat N, Panraksa K, Komalamisra C, et al Health Status of Immigrant Children and Environmental Survey of Child Daycare Centers in Samut Sakhon Province, Thailand. J Immigr Minor Health. 2016; 18: 21–27. 10.1007/s10903-014-0146-0 25502792

[6] Xiao L. Molecular epidemiology of cryptosporidiosis: an update. Exp Parasitol. 2010; 124: 80–89. 10.1016/j.exppara.2009.03.018 19358845

[7] Sim S, Won J, Kim JW, Kim K, Park WY, Yu JR. Simultaneous Molecular Detection of Cryptosporidium and Cyclospora from Raw Vegetables in Korea. Korean J Parasitol. 2017; 55: 137–142. 10.3347/kjp.2017.55.2.137 28506035 PMC5450956

[8] Abu-Madi MA, Behnke JM, Boughattas S, Al-Thani A, Doiphode SH. A decade of intestinal protozoan epidemiology among settled immigrants in Qatar. BMC Infect Dis. 2016; 16: 370 10.1186/s12879-016-1728-3 27496143 PMC4974681

[9] Abu-Madi M, Boughattas S, Behnke JM, Sharma A, Ismail A. Coproscopy and molecular screening for detection of intestinal protozoa. Parasit Vectors. 2017; 10: 414 10.1186/s13071-017-2346-7 28877704 PMC5588727

[10] Idris MA, Al-Jabri AM. Usefulness of Kato-Katz and trichrome staining as diagnostic methods for parasitic infections in clinical laboratories. J Sci Res Med Sci. 2001; 3: 65–68. 24019710 PMC3174709

[11] Sharif M, Daryani A, Kia E, Rezaei F, Nasiri M, Nasrolahei M. Prevalence of intestinal parasites among food handlers of Sari, Northern Iran. Rev Inst Med Trop Sao Paulo. 2015; 57: 139–144. 10.1590/S0036-46652015000200007 25923893 PMC4435012

[12] Zaglool DA, Khodari YA, Othman RA, Farooq MU. Prevalence of intestinal parasites and bacteria among food handlers in a tertiary care hospital. Niger Med J. 2011; 52: 266–270. 10.4103/0300-1652.93802 22529512 PMC3329099

[13] Duedu KO, Yarnie EA, Tetteh-Quarcoo PB, Attah SK, Donkor ES, Ayeh-Kumi PF. A comparative survey of the prevalence of human parasites found in fresh vegetables sold in supermarkets and open-aired markets in Accra, Ghana. BMC Res Notes. 2014; 7: 836 10.1186/1756-0500-7-836 25424391 PMC4253987

[14] Nazemalhosseini-Mojarad E, Feng Y, Xiao L. The importance of subtype analysis of Cryptosporidium spp. in epidemiological investigations of human cryptosporidiosis in Iran and other Mideast countries. Gastroenterol Hepatol Bed Bench. 2012; 5: 67–70. 24834202 PMC4017458

[15] Boughattas S, Behnke JM, Al-Ansari K, Sharma A, Abu-Alainin W, Al-Thani A, et al Molecular Analysis of the Enteric Protozoa Associated with Acute Diarrhea in Hospitalized Children. Front Cell Infect Microbiol. 2017; 7: 343 10.3389/fcimb.2017.00343 28824878 PMC5539595

[16] Coupe S, Sarfati C, Hamane S, Derouin F. Detection of cryptosporidium and identification to the species level by nested PCR and restriction fragment length polymorphism. J Clin Microbiol. 2005; 43: 1017–1023. 10.1128/JCM.43.3.1017-1023.2005 15750054 PMC1081268

[17] Sulaiman IM, Hira PR, Zhou L, Al-Ali FM, Al-Shelahi FA, Shweiki HM, et al Unique endemicity of cryptosporidiosis in children in Kuwait. J Clin Microbiol. 2005; 43: 2805–2809 10.1128/JCM.43.6.2805-2809.2005 15956401 PMC1151898

[18] Diaz P, Varcasia A, Pipia AP, Tamponi C, Sanna G, Prieto A, et al Molecular characterisation and risk factor analysis of Cryptosporidium spp. in calves from Italy. Parasitol Res. 2018; 14 10.1007/s00436-018-6000-x 30008134 PMC7088234

[19] Rohlf FJ, Sokal RR. Statistical Tables Freeman W.H. and Company, San Francisco; 1995.

[20] Janovy JJr, Clopton RE, Clopton DA, Snyder SD, Efting A. Krebs L. Species density distributions as null models for ecologically significant interactions of parasite species in an assemblage. Ecological Modelling. 1995; 77: 189–196.

[21] Balarak D, Modrek MJ, Bazrafshan E, Ansari H, Kord Mostafapour F. Prevalence of Intestinal Parasitic Infection among Food Handlers in Northwest Iran. J Parasitol Res. 2016; 2016: 8461965 10.1155/2016/8461965 27127643 PMC4834171

[22] Areeshi MY, Beeching NJ, Hart CA. Cryptosporidiosis in Saudi Arabia and neighboring countries. Ann Saudi Med. 2007; 27: 325–332. 10.5144/0256-4947.2007.325 17921688 PMC6077050

[23] Wang W, Cao L, He B, Li J, Hu T, Zhang F, et al Molecular characterisation of Cryptosporidium in bats from Yunnan province, southwestern China. J Parasitol. 2013; 99: 1148–1150. 10.1645/13-322.1 23886252

[24] Amer S, Harfoush M, He H. Molecular and phylogenetic analyses of Cryptosporidium SPP from dairy cattle in Egypt. J Egypt Soc Parasitol. 2010; 40: 349–366. 21246942

[25] Helmy YA, Krücken J, Nöckler K, von Samson-Himmelstjerna G, Zessin KH. Molecular epidemiology of Cryptosporidium in livestock animals and humans in the Ismailia province of Egypt. Vet Parasitol. 2013; 193: 15–24. 10.1016/j.vetpar.2012.12.015 23305974

[26] Ng J, MacKenzie B, Ryan U. Longitudinal multi-locus molecular characterisation of sporadic Australian human clinical cases of cryptosporidiosis from 2005 to 2008. Exp Parasitol. 2010; 125: 348–356. 10.1016/j.exppara.2010.02.017 20206624

[27] Iqbal J, Khalid N, Hira PR. Cryptosporidiosis in Kuwaiti children: association of clinical characteristics with Cryptosporidiumspecies and subtypes. J Med Microbiol. 2011; 60: 647–652. 10.1099/jmm.0.028001-0 21233297

[28] Alyousefi NA, Mahdy MA, Lim YA, Xiao L, Mahmud R. First molecular characterization of Cryptosporidium in Yemen. Parasitology. 2013; 140: 729–734. 10.1017/S0031182012001953 23369243

[29] Gherasim A, Lebbad M, Insulander M, Decraene V, Kling A, Hjertqvist M, et al Two geographically separated food-borne outbreaks in Sweden linked by an unusual Cryptosporidium parvum subtype, October 2010. Euro Surveill. 2012; 17: 20318 10.2807/ese.17.46.20318-en 23171824

[30] Taylan-Ozkan A, Yasa-Duru S, Usluca S, Lysen C, Ye J, Roellig DM, Feng Y, et al Cryptosporidium species and Cryptosporidium parvum subtypes in dairy calves and goat kids reared under traditional farming systems in Turkey. Exp Parasitol. 2016; 170: 16–20. 10.1016/j.exppara.2016.06.014 27373430

[31] Tao W, Li Y, Yang H, Song M, Lu Y, Li W. Widespread Occurrence of Zoonotic Cryptosporidium Species and Subtypes in Dairy Cattle from Northeast China: Public Health Concerns. J Parasitol. 2018; 104: 10–17. 10.1645/17-140 29088547

[32] Feng Y, Li N, Duan L, Xiao L. Cryptosporidium genotype and subtype distribution in raw wastewater in Shanghai, China: evidence for possible unique Cryptosporidium hominis transmission. J Clin Microbiol. 2009; 47: 153–157. 10.1128/JCM.01777-08 19005143 PMC2620847

[33] Gatei W, Barrett D, Lindo JF, Eldemire-Shearer D, Cama V, Xiao L. Unique Cryptosporidium population in HIV-infected persons, Jamaica. Emerg Infect Dis. 2008; 14: 841–843. 10.3201/eid1405.071277 18439378 PMC2600223

[34] Waldron LS, Ferrari BC, Cheung-Kwok-Sang C, Beggs PJ, Stephens N, Power ML. Molecular epidemiology and spatial distribution of a waterborne cryptosporidiosis outbreak in Australia. Appl Environ Microbiol. 2011; 77: 7766–7771. 10.1128/AEM.00616-11 21908623 PMC3209151

[35] Koehler AV, Whipp M, Hogg G, Haydon SR, Stevens MA, Jex AR, et al First genetic analysis of Cryptosporidium from humans from Tasmania, and identification of a new genotype from a traveler to Bali. Electrophoresis. 2014; 35: 2600–2607. 10.1002/elps.201400225 24916177

[36] Hatalova E, Valencakova A, Luptakova L, Spalkova M, Kalinova J, Halanova M, et al The first report of animal genotypes of Cryptosporidium parvum in immunosuppressed and immunocompetent humans in Slovakia. Transbound Emerg Dis. 2019; 66: 243–249. 10.1111/tbed.13009 30179310

[37] Stensvold CR, Beser J, Axén C, Lebbad M. High applicability of a novel method for gp60-based subtyping of Cryptosporidium meleagridis. J Clin Microbiol. 2014; 52: 2311–2319. 10.1128/JCM.00598-14 24740082 PMC4097674

[38] Baroudi D, Khelef D, Goucem R, Adjou KT, Adamu H, Zhang H, et al Common occurrence of zoonotic pathogen Cryptosporidium meleagridis in broiler chickens and turkeys in Algeria. Vet Parasitol. 2013; 196: 334–340. 10.1016/j.vetpar.2013.02.022 23498647

[39] Lee JD, Chiu ML, Chung LY, Yen CM. Prevalence of Cryptosporidium for foreign workers in Taiwan. Trop Doct. 2004; 34: 185–186. 10.1177/004947550403400325 15267060

[40] Sahimin N, Douadi B, Yvonne Lim AL, Behnke JM, Mohd Zain SN. Distribution of Giardia duodenalis (Assemblages A and B) and Cryptosporidium parvum amongst migrant workers in Peninsular Malaysia. Acta Trop. 2018; 182: 178–184. 10.1016/j.actatropica.2018.02.033 29501402

[41] Ghenghesh KS, Ghanghish K, El-Mohammady H, Franka E. Cryptosporidium in countries of the Arab world: the past decade (2002–2011). Libyan J Med. 2012; 7 10.3402/ljm.v7i0.19852 23198000 PMC3509416

[42] Chalmers RM, Katzer F. Looking for Cryptosporidium: the application of advances in detection and diagnosis. Trends Parasitol. 2013; 29: 237–251. 10.1016/j.pt.2013.03.001 23566713 PMC7106352

[43] Soriano SV, Pierangeli NB, Roccia I, Bergagna HF, Lazzarini LE, Celescinco A, et al A wide diversity of zoonotic intestinal parasites infects urban and rural dogs in Neuquén, Patagonia, Argentina. Vet Parasitol. 2010; 167: 81–85. 10.1016/j.vetpar.2009.09.048 19864068

[44] Omoruyi B, Matongo F, Nkwetshana NT, Green E, Clarke AM, Ndip RN. Environmental and demographic risk factors associated with the prevalence of Cryptosporidium infection in the Alice rural settlements of the Eastern Cape Province of South Africa: a pilot study. Rev Environ Health. 2011; 26: 127–133. 21905456 10.1515/reveh.2011.017

[45] Moore CE, Elwin K, Phot N, Seng C, Mao S, Suy K, et al Molecular Characterisation of Cryptosporidium Species and Giardia duodenalis from Symptomatic Cambodian Children. PLoS Negl Trop Dis. 2016;10: e0004822 10.1371/journal.pntd.0004822 27387755 PMC4936737

[46] Nawalinski T, Schad GA, Chowdhury AB. Population biology of hookworms in children in rural West Bengal. II. Acquisition and loss of hookworms. Am J Trop Med Hyg. 1978; 27: 1162–1173. 10.4269/ajtmh.1978.27.1162 727321

[47] Gelaw A, Anagaw B, Nigussie B, Silesh B, Yirga A, Alem M, et al Prevalence of intestinal parasitic infections and risk factors among schoolchildren at the University of Gondar Community School, Northwest Ethiopia: a cross-sectional study. BMC Public Health. 2013; 13: 304 10.1186/1471-2458-13-304 23560704 PMC3621079

[48] Quihui-Cota L, Lugo-Flores CM, Ponce-Martínez JA, Morales-Figueroa GG. Cryptosporidiosis: a neglected infection and its association with nutritional status in schoolchildren in northwestern Mexico. J Infect Dev Ctries.https://www.ncbi.nlm.nih.gov/pubmed/26322881 2015; 9: 878–883. 10.3855/jidc.6751 26322881

[49] Becker DJ, Oloya J, Ezeamama AE. Household Socioeconomic and Demographic Correlates of Cryptosporidium Seropositivity in the United States. PLoS Negl Trop Dis. 2015; 9: e0004080 10.1371/journal.pntd.0004080 26368018 PMC4569081

[50] Chacín-Bonilla L, Barrios F, Sanchez Y. Environmental risk factors for Cryptosporidium infection in an island from Western Venezuela. Mem Inst Oswaldo Cruz. 2008; 103: 45–49. 10.1590/s0074-02762008005000007 18345459

[51] Daniels ME, Smith WA, Schmidt WP, Clasen T, Jenkins MW. Modeling Cryptosporidium and Giardia in Ground and Surface Water Sources in Rural India: Associations with Latrines, Livestock, Damaged Wells, and Rainfall Patterns. Environ Sci Technol. 2016; 50: 7498–7507. 10.1021/acs.est.5b05797 27310009 PMC5058636

[52] Sahimin N, Lim YA, Ariffin F, Behnke JM, Lewis JW, Mohd Zain SN. Migrant Workers in Malaysia: Current Implications of Sociodemographic and Environmental Characteristics in the Transmission of Intestinal Parasitic Infections. PLoS Negl Trop Dis. 2016; 10: e0005110 10.1371/journal.pntd.0005110 27806046 PMC5091761

[53] Sarkari B, Hosseini G, Motazedian MH, Fararouei M, Moshfe A. Prevalence and risk factors of intestinal protozoan infections: a population-based study in rural areas of Boyer-Ahmad district, Southwestern Iran. BMC Infect Dis. 2016; 16:703 10.1186/s12879-016-2047-4 27884121 PMC5123427

[54] Nematian J, Nematian E, Gholamrezanezhad A, Asgari AA. Prevalence of intestinal parasitic infections and their relation with socio-economic factors and hygienic habits in Tehran primary school students. Acta Trop. 2004; 92: 179–186. 10.1016/j.actatropica.2004.06.010 15533285

